# Effects of Enhanced Recovery After Surgery-Based Nursing on Kinesiophobia and Anxiety-Depression in Elderly Patients After Hip Arthroplasty: A Retrospective Cohort Study

**DOI:** 10.62641/aep.v54i3.2191

**Published:** 2026-06-15

**Authors:** Zheng Tian, Huan Liu, Lu Yang, Hehuan Xia, Yanjiao Li

**Affiliations:** ^1^Department of Orthopaedic Surgery, Hebei Medical University Third Hospital, 050000 Shijiazhuang, Hebei, China

**Keywords:** enhanced postsurgical recovery, nursing, hip arthroplasty, anxiety, depression

## Abstract

**Background:**

Elderly patients undergoing hip arthroplasty frequently experience postoperative pain, limited mobility, and negative psychological states, which adversely affect rehabilitation outcomes. This study aimed to investigate the effect of Enhanced Recovery After Surgery (ERAS) on kinesiophobia, anxiety, and depression in elderly patients after hip arthroplasty.

**Methods:**

A single-centre retrospective cohort study of 132 elderly patients who underwent hip arthroplasty was conducted. Relevant data were extracted from the hospital medical record system. Based on the documented nursing approach, patients were classified into a control group that received routine nursing and a study group that received ERAS-based nursing. Kinesiophobia, anxiety, depression, hope level, hip function, health-promoting lifestyle, pain intensity, and quality of life were assessed using Tampa Scale for Kinesiophobia (TSK), Hamilton Anxiety Scale (HAMA), 17-item Hamilton Depression Scale (HAMD-17), Herth Hope Index (HHI), Harris Hip Score (HHS), Health-Promoting Lifestyle Profile II (HPLP-II), and Visual Analogue Scale (VAS), as recorded in the medical records before and after nursing intervention.

**Results:**

There were no significant differences in baseline characteristics or pre-nursing care scores between the two groups (*p* > 0.05). After nursing care, both groups showed significant improvements in all outcomes (*p* < 0.05). Specifically, the study group demonstrated significantly lower TSK, HAMA, HAMD-17, and VAS scores and higher HHI, HHS, and HPLP-II scores compared with the control group (*p* < 0.05).

**Conclusions:**

The findings of this study suggest that ERAS-based nursing may alleviate negative emotions, reduce pain, promote functional recovery, and improve quality of life in elderly patients after hip arthroplasty.

## Introduction

Hip arthroplasty is a commonly used and effective treatment for degenerative hip diseases, femoral neck fractures, and severe hip joint dysfunction in elderly patients, and it can remarkably relieve pain, improve joint function, and enhance quality of life [1, 2]. However, due to age-related physiological decline, a high prevalence of comorbidities, and insufficient understanding of surgery and rehabilitation, elderly patients often experience varying degrees of postoperative pain, limited mobility, and psychological stress responses, particularly kinesiophobia, anxiety, and depression [3, 4, 5]. These psychological disorders not only reduce patient adherence to functional rehabilitation exercises but may also delay the recovery process, thereby adversely affecting hip function restoration [6].

Kinesiophobia refers to an excessive fear of movement and avoidance behaviour caused by patients’ concerns about aggravating pain or sustaining re-injury, and it is relatively common among elderly patients after hip arthroplasty [7, 8]. Studies have shown that kinesiophobia is closely associated with postoperative activity limitation and poor functional recovery. Meanwhile, negative emotions such as anxiety and depression may interact with kinesiophobia, forming a vicious cycle [9]. In addition, positive psychological resources such as hope level and health-promoting lifestyle may influence recovery, but these factors have been relatively underexplored in elderly patients undergoing hip arthroplasty. Therefore, how to alleviate kinesiophobia and improve anxiety and depressive symptoms through effective perioperative nursing is a crucial nursing issue for promoting rapid recovery in elderly patients after hip arthroplasty.

Enhanced Recovery After Surgery (ERAS) emphasises optimising perioperative management through multidisciplinary collaboration and evidence-based nursing measures, reducing surgical stress responses, and promoting early mobilisation and functional recovery [10, 11]. In recent years, the ERAS concept has been increasingly applied in the field of orthopaedics, and its effectiveness in shortening hospital stay and reducing the incidence of complications has been well documented [12, 13, 14]. However, the previous study has primarily focused on physiological outcomes such as pain, complications, and length of stay [15]. The effects of ERAS-based nursing on psychological and behavioural outcomes, particularly kinesiophobia and hope level, remain insufficiently studied, especially in elderly hip arthroplasty patients [16]. Based on this gap, the present retrospective study specifically aimed to examine the impact of ERAS-based nursing on both negative psychological outcomes (kinesiophobia, anxiety, and depression) and positive psychological factors (hope level and health-promoting lifestyle), as well as functional recovery, pain intensity, and quality of life, in elderly patients undergoing hip arthroplasty. By integrating physical, psychological, and behavioural outcomes, this study seeks to extend existing evidence on ERAS in orthopaedic populations and provide data-driven support for optimising perioperative nursing strategies in this vulnerable population.

## Materials and Methods

### General Information

This study was a single-centre retrospective study. A total of 132 elderly patients who underwent hip arthroplasty in our hospital between January 2025 and December 2025 were retrospectively identified from the hospital medical record system. According to the nursing model recorded in the patient files, the patients were divided into a control group (n = 63) and a study group (n = 69). The decision regarding whether a patient received ERAS-based nursing or routine nursing was not based on individual patient characteristics, but rather on the standardised perioperative nursing protocol adopted by the department during different periods. The general characteristics of the two groups are presented in Table 1.

**Table 1.** **Baseline characteristics of patients in the two groups**.

Factors		Control group (n = 63)	Study group (n = 69)	t/χ^2^	*p*
Gender					
	Male	31 (49.21)	33 (47.83)	0.025	0.874
	Female	32 (50.79)	36 (52.17)		
Age (years old)		67.67 ± 3.98	67.92 ± 4.12	0.353	0.725
BMI (kg/m^2^)		22.38 ± 0.54	22.45 ± 0.52	0.754	0.452
Type of arthroplasty					
	Hemiarthroplasty	10 (15.87)	14 (20.29)	0.432	0.511
	Total hip arthroplasty	53 (84.13)	55 (79.71)		
ASA Classification					
	ASA I	30 (47.62)	32 (46.38)	0.020	0.886
	ASA II	33 (52.38)	37 (53.62)		
Surgery duration (h)		1.32 ± 0.12	1.35 ± 0.14	1.337	0.184
Intraoperative blood loss (mL)		267.46 ± 14.35	265.41 ± 15.85	0.777	0.438

BMI, body mass index; ASA, American Society of Anesthesiologists.

The inclusion criteria were as follows: (1) patients aged ≥60 years who underwent hip arthroplasty in our hospital; (2) able to tolerate anaesthesia; (3) received complete perioperative and postoperative nursing with clearly defined nursing models (ERAS-based nursing or routine nursing); (4) had basic communication and comprehension abilities and were able to cooperate with assessments using scales for kinesiophobia, anxiety, and depression; and (5) complete clinical data with available postoperative follow-up and relevant evaluation data.

The exclusion criteria were as follows: (1) severe dysfunction of vital organs (heart, brain, lungs, liver, or kidneys) that substantially increased perioperative risk or interfered with standardised ERAS implementation and postoperative rehabilitation assessment; (2) severe cognitive impairment, dementia, or other psychiatric conditions that prevented reliable completion of psychological and functional scale assessments; (3) a clearly diagnosed severe anxiety, depression, or other major psychiatric disorder prior to surgery and receipt of systematic psychiatric treatment, which could substantially influence baseline psychological evaluation; (4) malignant tumours or other serious conditions associated with limited life expectancy that might affect postoperative recovery and quality-of-life assessment; (5) development of severe perioperative complications (e.g., severe infection, reoperation, major cardiovascular or cerebrovascular events) that substantially interfered with postoperative rehabilitation evaluation; and (6) incomplete clinical data or missing key follow-up information required for outcome analysis.

### Nursing Methods

#### Nursing Methods for the Control Group

Patients in the control group received routine perioperative nursing care [17]. Preoperatively, nursing staff assisted patients in completing relevant examinations and provided general health education, including an explanation of the surgical procedure and related precautions. Postoperatively, patients’ vital signs, surgical incision, and drainage were closely monitored. Analgesic, anti-infective, and other symptomatic supportive treatments were administered according to medical orders. Patients were instructed to maintain proper positioning to prevent complications such as deep vein thrombosis and pressure ulcers. When pain was alleviated and the patient’s condition permitted, routine functional exercise guidance was provided, including ankle pump exercises in bed and isometric quadriceps contractions, with gradual assistance for ambulation out of bed. During the nursing process, attention was paid to patients’ emotional changes, and basic psychological comfort and support were offered. This study was approved by the Ethics Committee of Hebei Medical University Third Hospital (Approval No.: W2025-023-1). All procedures involving human participants were conducted in accordance with the ethical standards of the institutional and/or national research committee and with the Declaration of Helsinki. All eligible participants signed an informed consent form.

#### Nursing Methods for the Study Group

Based on routine nursing care, patients in the study group received ERAS-guided nursing covering the preoperative, postoperative, and follow-up phases [18].

Preoperatively, comprehensive assessments of physical condition, functional status, and psychological state were conducted within 24–48 hours of admission. Structured health education was provided to patients and their families regarding the surgical procedure, ERAS principles, and postoperative rehabilitation plans, with emphasis on early mobilisation and strategies to alleviate anxiety and kinesiophobia.

Postoperatively, multimodal analgesia was implemented, and pain was dynamically assessed every 2–4 hours within the first 24 hours. Early functional exercises were initiated 6–12 hours after surgery when clinically stable, progressing from in-bed movements to bedside standing and assisted ambulation. Exercise intensity was gradually increased according to individual tolerance. Continuous psychological support was provided to reduce kinesiophobia, anxiety, and depressive symptoms, and patients and families were encouraged to actively participate in rehabilitation.

Before discharge, standardised home-based rehabilitation guidance was provided, and regular follow-up was conducted to monitor recovery progress, reinforce exercise adherence, and ensure continuity of care.

To ensure consistency, the ERAS-based nursing intervention was implemented according to a standardised nursing pathway developed by our department prior to the study. All participating nurses received unified training on ERAS principles and operational procedures. Key components—including early mobilisation timing, pain assessment frequency (every 2–4 hours within the first 24 hours), multimodal analgesia, and psychological support—were carried out according to predefined schedules and documented in standardised nursing records and the electronic medical system. Regular supervision and quality control were conducted by senior nursing staff to minimise inter-nurse variability and ensure protocol adherence.

Nursing interventions in both groups were initiated within 24 hours after admission and continued until discharge. The overall intervention period corresponded to the length of hospitalisation, with no statistically significant difference in hospital stay between the two groups (*p* > 0.05). In addition, both groups received perioperative nursing interventions according to a standardised frequency and protocol, ensuring consistency in the initiation time, termination point, and total duration of the intervention. Outcomes were assessed at identical time points in both groups: baseline evaluation was performed within 24 hours after admission, and post-intervention assessment was conducted on postoperative day seven.

### Outcome Measures

All patients in both groups were evaluated before (within 24 hours after admission) and after (postoperative day seven) the nursing care.

Kinesiophobia was assessed using the Tampa Scale for Kinesiophobia (TSK) [19, 20]. The scale consists of 17 items covering two domains: activity avoidance and fear of injury. Each item is rated on a 4-point Likert scale (1–4 points), with a total score ranging from 17 to 68 points; higher scores indicate more severe kinesiophobia. In the present study, the reliability and validity of the TSK were evaluated based on our study population. The internal consistency of the TSK demonstrated good reliability, with a Cronbach’s α coefficient of 0.78.

Anxiety and depression were assessed using the Hamilton Anxiety Scale (HAMA) [21] and the 17-item Hamilton Depression Scale (HAMD-17) [22], respectively. The HAMA includes 14 items encompassing psychic anxiety and somatic anxiety, with each item scored from 0 to 4 and a total score ranging from 0 to 56. The HAMD-17 contains 17 items mainly assessing mood, sleep, and somatic symptoms; most items are scored from 0 to 4, while a few are scored from 0 to 2. The total score range of the HAMD-17 is 0 to 52, with higher scores indicating more severe depressive symptoms. In the present study, the reliability of the HAMA was evaluated in our study population. The results demonstrated good internal consistency, with a Cronbach’s α coefficient of 0.87, indicating satisfactory reliability in the current sample. In the present study, the reliability of the HAMD-17 was assessed in our study population. The results showed good internal consistency, with a Cronbach’s α coefficient of 0.86, indicating satisfactory reliability in the current sample.

Hope level was measured using the Herth Hope Index (HHI) [23]. The HHI consists of 12 items across three dimensions—future expectations, relationships with others, and coping ability for real-life problems—each containing four items. A 4-point Likert scale (1–4 points) is used, with total scores ranging from 12 to 48; higher scores reflect higher levels of hope. In the present study, the reliability of the HHI was evaluated in our study population. The results demonstrated excellent internal consistency, with a Cronbach’s α coefficient of 0.97.

Hip function was evaluated using the Harris Hip Score (HHS) [24], which assesses pain, function, deformity, and range of motion. The total score ranges from 0 to 100, with higher scores indicating better hip function. In the present study, the reliability of the scale was assessed in our study population. The internal consistency, as measured by Cronbach’s α, was 0.82.

Health-promoting lifestyle was assessed using the Health-Promoting Lifestyle Profile II (HPLP-II) [25, 26]. This scale includes 52 items across six dimensions: health responsibility, physical activity, nutrition, interpersonal relations, spiritual growth, and stress management. Each item is rated on a 4-point Likert scale (1–4 points), yielding a total score range of 52 to 208; higher scores indicate a higher level of health-promoting lifestyle. In the present study, the reliability of the scale was evaluated in our study population. The results showed good internal consistency, with a Cronbach’s α coefficient of 0.89 for the total scale.

Pain intensity was evaluated using the Visual Analogue Scale (VAS) [27], with scores ranging from 0 to 10, where 0 indicates no pain and 10 indicates the most severe pain. In the present study, the reliability of the VAS was evaluated in our study population. The VAS demonstrated good internal consistency, with a Cronbach’s α coefficient of 0.83, indicating satisfactory reliability for assessing pain intensity in the current sample.

### Statistical Analysis

Statistical analyses were performed using SPSS (version 27.0, IBM, Armonk, NY, USA). Categorical variables were expressed as n(%) and analysed using the chi-square (χ^2^) test. Continuous variables were first tested for normality. Data conforming to a normal distribution were analysed using the independent-samples t test. After testing, all continuous variables in this study were found to conform to a normal distribution. Continuous variables were expressed as mean ± standard deviation (Mean ± SD). A *p* value < 0.05 was considered statistically significant.

## Results

### Comparison of General Characteristics Between the Two Groups

According to different nursing approaches, the patients were divided into a control group and a study group. There were no statistically significant differences between the two groups in general characteristics, including sex, age, body mass index (BMI), type of arthroplasty, ASA classification, operative time, and intraoperative blood loss (*p* > 0.05).

### Comparison of Kinesiophobia, Anxiety, and Depression between the Two Groups

Before the nursing, there were no statistically significant differences in TSK, HAMA, or HAMD-17 scores between the control group and the study group (*p* > 0.05). After the nursing, TSK, HAMA, and HAMD-17 scores in both groups were lower than those before the nursing. Moreover, after the nursing, the TSK, HAMA, and HAMD-17 scores in the study group were significantly lower than those in the control group, with statistically significant differences (*p* < 0.05) (Table 2).

**Table 2.** **Comparison of TSK, HAMA and HAMD-17 scores between the two groups**.

Factors	Control group (n = 63)	Study group (n = 69)	t/χ^2^	*p*
Pre-nursing TSK score	44.98 ± 4.54	45.11 ± 4.59	0.173	0.863
Post-nursing TSK score	39.56 ± 3.58	33.09 ± 3.66	10.260	<0.001
Pre-nursing HAMA score	19.07 ± 2.12	19.05 ± 2.09	0.051	0.960
Post-nursing HAMA score	14.16 ± 1.74	11.57 ± 1.61	8.873	<0.001
Pre-nursing HAMD-17 score	19.50 ± 2.00	19.43 ± 1.93	0.204	0.838
Post-nursing HAMD-17 score	14.56 ± 1.79	12.29 ± 1.76	7.330	<0.001

TSK, Tampa Scale for Kinesiophobia; HAMA, Hamilton Anxiety Scale; HAMD, Hamilton Depression Scale.

### Comparison of Hope Levels between the Two Groups

Before the nursing, there were no statistically significant differences in the HHI scores (including three dimensions: future expectations, relationships with others, coping ability for real-life problems, and the total score) between the control group and the study group (*p* > 0.05). After the nursing, HHI scores in both groups were higher than those before the nursing. Moreover, after the nursing, HHI scores in the study group were significantly higher than those in the control group, with statistically significant differences (*p* < 0.05) (Table 3).

**Table 3.** **Comparison of HHI scores between the two groups**.

Factors	Control group (n = 63)	Study group (n = 69)	t/χ^2^	*p*
Pre-nursing future expectations	8.24 ± 0.92	8.17 ± 0.87	0.439	0.662
Post-nursing future expectations	9.19 ± 1.16	10.50 ± 1.20	6.388	<0.001
Pre-nursing relationships with others	8.39 ± 1.04	8.33 ± 1.01	0.339	0.735
Post-nursing relationships with others	9.27 ± 1.11	10.49 ± 1.25	5.924	<0.001
Pre-nursing coping ability for real-life problems	8.69 ± 1.00	8.83 ± 0.93	0.823	0.412
Post-nursing coping ability for real-life problems	9.79 ± 1.13	11.20 ± 1.11	7.222	<0.001
Pre-nursing total score	25.30 ± 2.19	25.13 ± 2.17	0.453	0.651
Post-nursing total score	28.39 ± 2.39	32.06 ± 2.67	8.286	<0.001

### Comparison of Hip Function and Health-Promoting Lifestyle Levels between the Two Groups

Before the nursing, there were no statistically significant differences in HHS or HPLP-II scores between the two groups (*p* > 0.05). After the nursing, HHS and HPLP-II scores in both groups were higher than those before the nursing. Moreover, after the nursing, the HHS and HPLP-II scores in the study group were significantly higher than those in the control group, with statistically significant differences (*p* < 0.05) (Table 4).

**Table 4.** **Comparison of HHS and HPLP-II scores between the two groups**.

Factors	Control group (n = 63)	Study group (n = 69)	t/χ^2^	*p*
Pre-nursing HHS score	50.95 ± 5.27	50.96 ± 5.40	0.008	0.994
Post-nursing HHS score	70.96 ± 7.61	79.46 ± 8.03	6.225	<0.001
Pre-nursing HPLP-II score	74.81 ± 7.73	75.24 ± 7.56	0.328	0.744
Post-nursing HPLP-II score	152.04 ± 16.85	168.52 ± 16.87	5.608	<0.001

HHS, Harris Hip Score; HPLP-II, Health-Promoting Lifestyle Profile II.

### Comparison of Pain Levels and Quality of Life between the Two Groups

Before the nursing, there were no statistically significant differences between the two groups in VAS scores (*p* > 0.05). After the nursing, VAS scores in both groups were lower than those before the nursing. Moreover, after the nursing, the VAS score in the study group was significantly lower than that in the control group, with a statistically significant difference (*p* < 0.05) (Table 5).

**Table 5.** **Comparison of VAS scores between the two groups**.

Factors	Control group (n = 63)	Study group (n = 69)	t/χ^2^	*p*
Pre-nursing VAS score	7.45 ± 1.05	7.50 ± 1.08	0.277	0.782
Post-nursing VAS score	4.49 ± 0.81	3.05 ± 0.74	10.716	<0.001

VAS, Visual Analogue Scale.

## Discussion

The results of this study showed that there were no statistically significant differences between the two groups in terms of general characteristics, including sex, age, BMI, type of arthroplasty, ASA (American Society of Anesthesiologists) classification, operative time, and intraoperative blood loss, indicating good baseline comparability between the groups and providing a reliable basis for comparing the effects of the nursing.

In terms of kinesiophobia, anxiety, and depression, this study found that after ERAS-based nursing, the TSK, HAMA, and HAMD-17 scores in the study group were significantly lower than those in the control group. These findings are generally consistent with previous studies on the impact of ERAS nursing on perioperative psychological outcomes in surgical patients [28]. Existing evidence suggests that systematic health education, adequate pain control, and early safe mobilisation are key factors in alleviating postoperative negative emotions and kinesiophobia, as they effectively enhance patients’ sense of control and safety during the rehabilitation process [29, 30]. The present study further confirms the positive role of ERAS-based nursing in reducing kinesiophobia as well as anxiety and depressive symptoms in elderly patients undergoing hip arthroplasty, with superior effects compared with conventional nursing care.

Hope level, as an important indicator reflecting patients’ psychological resilience and beliefs regarding recovery, has received increasing attention in nursing research in recent years [31, 32]. The results of this study showed that, after the nursing, patients in the study group had significantly higher scores across all dimensions of the HHI compared with those in the control group. A relevant study suggests that participatory nursing models and the establishment of clear rehabilitation goals can enhance patients’ expectations of recovery outcomes and their initiative in rehabilitation, thereby improving hope levels [33]. The present findings further indicate that integrating the ERAS concept into nursing care can help improve the positive psychological status of elderly patients undergoing hip arthroplasty.

Regarding the recovery of hip joint function, this study demonstrated that the post-nursing HHS scores in the study group were significantly higher than those in the control group. The previous study has confirmed that the ERAS concept, by emphasising early postoperative mobilisation and progressive functional training, can promote joint function recovery and shorten the rehabilitation period [34]. The results of the present study are consistent with these findings, suggesting that the implementation of ERAS-based nursing in elderly patients undergoing hip arthroplasty not only facilitates early functional improvement but also indirectly promotes functional recovery by alleviating kinesiophobia and negative emotional states.

Regarding health-promoting lifestyles, this study found that the post-nursing HPLP-Ⅱ scores in the study group were significantly higher than those in the control group. These findings further indicate that ERAS-based nursing not only focuses on short-term functional outcomes but also, to a certain extent, promotes long-term improvement in patients’ health behaviours.

Pain control is an important indicator for evaluating postoperative recovery after hip arthroplasty. The results of this study showed that, after the nursing, the VAS scores of patients in the study group were significantly lower than those in the control group. This may be attributed to the combined effects of multimodal analgesia in reducing pain perception, early mobilisation in improving physiological function, and improvements in psychological status, which together enhance overall quality of life across physical, psychological, and social domains [35].

This study has several limitations. First, as a single-centre retrospective study with a relatively small sample size, the generalizability of the findings are limited, and selection bias cannot be excluded due to the non-randomized group allocation. Although baseline characteristics were comparable, unmeasured confounders—such as patient motivation, family support, nursing staff experience, and perioperative analgesic strategies—may have influenced the outcomes. In addition, the relatively short follow-up period prevented further evaluation of the long-term effects of ERAS-based nursing on psychological status and functional recovery. Therefore, future multicentre, large-sample prospective randomized controlled trials, incorporating methods such as propensity score matching or multivariable adjustment, are needed to validate the long-term efficacy and broader applicability of ERAS-based nursing. Although the ERAS protocol was standardised and supervised, detailed quantitative analysis of compliance with each ERAS component was not separately performed. Therefore, the relative contribution of individual ERAS elements to the observed outcomes could not be fully determined, which represents a methodological limitation of the present study. A further limitation is that the statistical analysis relied on unadjusted between-group comparisons despite the use of multiple correlated outcome measures, which may increase the risk of type I error. No correction for multiple comparisons was applied, potentially affecting the robustness of some significant findings. Future studies should incorporate appropriate adjustment methods or multivariable analyses to improve statistical rigor.

## Conclusions

In summary, the findings of this study are generally consistent with previous reports and further confirm, in elderly patients undergoing hip arthroplasty, the comprehensive advantages of ERAS-based nursing in improving kinesiophobia, anxiety and depression, hope levels, functional recovery, health-promoting behaviours, pain control, and quality of life, thereby providing evidence-based support for its wider implementation in clinical nursing practice.

## Availability of Data and Materials

The data that support the findings of this study are available from the corresponding author upon reasonable request.
